# A changed world built on informatics innovation

**DOI:** 10.1093/jamiaopen/ooab002

**Published:** 2021-02-12

**Authors:** Indra Neil Sarkar

**Affiliations:** 1 Rhode Island Quality Institute, Providence, Rhode Island, USA; 2 Center for Biomedical Informatics; Brown University, Providence, Rhode Island, USA

It is simply astounding to consider the substantial advancements of biomedical informatics over its short 60-year history. Many will recall the era where paper charts dominated the practice of medicine and access to electronic health records was largely relegated to the precursor to what we now refer to as an academic health center. Along with the exponential advancements in digital technologies, led by the advent and rapid availability of microcomputers (what most now refer to as just a computer), the concept of digital medicine went from scholarly pursuit to clinical requirement. It is rare now to find a clinician who does not interact with a digital system, whether it be a simple electronic health record system to reference information about a patient or a more advanced clinical decision support system to guide a decision, to provide the highest quality of care. There are still many areas where refinement and improvement are needed, which form the basis of the content for *JAMIA Open* articles.

The predominant vision for biomedical informaticians has long been to develop, implement, and evaluate solutions that would enable a digital future for medicine through the provision of the right electronic health information to the right person at the right time. In the past year, the spread of the SARS-CoV-2 virus resulted in the COVID-19 pandemic, we saw increased reliance on digital systems to support clinical and public health needs. There were, and continue to be, studies specific to how electronic systems have been developed or adapted to support diagnosis, treatment, and now vaccination for COVID-19. While COVID-19 dominated the headlines, advances across the full spectrum of health care continued. As with many biomedical journals, *JAMIA Open* did prioritize COVID-19–related manuscripts through its peer-review process without sacrificing scholarly integrity or scientific quality. It is our intention to continue to do so as long as the pandemic results in record numbers of individuals being diagnosed, hospitalized, or dying from COVID-19 or its sequalae.

Even during a pandemic of historic proportions, healthcare challenges that existed before still remain. The research community continues its foundational work of improving models to better understand disease mechanisms, advancing approaches to support integration and use of electronic health data, or demonstrating how the latest algorithms can measurably increase the diagnosis, treatment, or monitoring of disease. The constant flow of submissions to *JAMIA Open* reflects this, and with each issue extending our overall toolkit for transforming data into actionable clinical insights. As you read and experience the science presented across this issue, you will find a range of articles that reflect the breadth of biomedical informatics. We are fortunate that the regular submissions enable the development of issues that have something for the entirety of the biomedical informatics community.

Having concluded our first major phase to launch *JAMIA Open*, I am pleased to reflect on the success that the journal has had as a result of the submissions, reviewers, and growing readership. We were purposeful in allowing the scope of the journal to develop organically, which has resulted in a strong and synergistic partnership with our sibling journal, *JAMIA*. Starting next year, we will begin the introduction of a limited number of special issues focused on specific topic areas. If you would be interested in leading, in collaboration with our editorial team, a topical special issue, look for more specific instructions in the coming months.

We will also be focusing this coming year on increasing our community of readers. As a young journal, our overall impact and readership depend on each share, tweet, and reference for both our short-term success and long-term sustainability. Please take a few moments to share your reflections on your favorite articles from this issue on your social media!

As always, I thank you for your support for *JAMIA Open* and hope you enjoy this latest issue.

